# Novel Activity Detection Algorithm to Characterize Spontaneous Stepping During Multimodal Spinal Neuromodulation After Mid-Thoracic Spinal Cord Injury in Rats

**DOI:** 10.3389/fnsys.2019.00082

**Published:** 2020-01-15

**Authors:** Raymond Chia, Hui Zhong, Bryce Vissel, V. Reggie Edgerton, Parag Gad

**Affiliations:** ^1^Faculty of Science, Centre for Neuroscience and Regenerative Medicine, University of Technology Sydney, Sydney, NSW, Australia; ^2^St Vincent’s Centre for Applied Medical Research, Sydney, NSW, Australia; ^3^Department of Integrative Biology and Physiology, University of California, Los Angeles, Los Angeles, CA, United States; ^4^Department of Neurobiology, University of California, Los Angeles, Los Angeles, CA, United States; ^5^Department of Neurosurgery, University of California, Los Angeles, Los Angeles, CA, United States; ^6^Brain Research Institute, University of California, Los Angeles, Los Angeles, CA, United States; ^7^Institut Guttmann, Hospital de Neurorehabilitació, Institut Universitari Adscrit a la Universitat Autònoma de Barcelona, Barcelona, Spain

**Keywords:** sub-threshold spinal cord stimulation, spinal cord injury, spontaneous cage activity, EMG, muscle coordination, strychnine, quipazine, evoked potentials

## Abstract

A mid-thoracic spinal cord injury (SCI) severely impairs activation of the lower limb sensorimotor spinal networks, leading to paralysis. Various neuromodulatory techniques including electrical and pharmacological activation of the spinal networks have been successful in restoring locomotor function after SCI. We hypothesized that the combination of self-training in a natural environment with epidural stimulation (ES), quipazine (Quip), and strychnine (Strych) would result in greater activity in a cage environment after paralysis compared to either intervention alone. To assess this, we developed a method measuring and characterizing the chronic EMG recordings from tibialis anterior (TA) and soleus (Sol) muscles while rats were freely moving in their home cages. We then assessed the relationship between the change in recorded activity over time and motor-evoked potentials (MEPs) in animals receiving treatments. We found that the combination of ES, Quip, and Strych (sqES) generated the greatest level of recovery followed by ES + Quip (qES) while ES + Strych (sES) and ES alone showed least improvement in recorded activity. Further, we observed an exponential relationship between late response (LR) component of the MEPs and spontaneously generated step-like activity. Our data demonstrate the feasibility and potential importance of quantitatively monitoring mechanistic factors linked to activity-dependence in response to combinatorial interventions compared to individual therapies after SCI.

## Introduction

Numerous studies of different animal models of spinal cord injury (SCI) have demonstrated that modulating the physiological states of spinal networks, pharmacologically and electrically and in combination with motor training enables improved motor performance (Edgerton et al., 2004; Ichiyama et al., 2005; Gerasimenko et al., 2008; Lavrov et al., 2008; Courtine et al., 2009; Harkema et al., 2011; Rossignol and Frigon, 2011; Gad et al., 2013a, b; Angeli et al., 2014; Gerasimenko Y. et al., 2015; Capogrosso et al., 2016; Formento et al., 2018; Gill et al., 2018). Specific pharmacology methods including the use of quipazine (Quip) (Fong et al., 2005; Ichiyama et al., 2008b; Gad et al., 2015; Taccola et al., 2018) and strychnine (Strych) (de Leon et al., 1999; Gad et al., 2013b, 2015) have been effective in activation in the locomotor spinal neural networks to enable locomotor activity in rodent with severe paralysis. Similar interventional approaches have been observed in humans with a severe spinal injury (Gerasimenko Y. et al., 2015; Gerasimenko Y. P. et al., 2015). Recently, we explored the effects of long-term sub-motor threshold ES in rats with complete mid-thoracic spinal transections (Gad et al., 2013a). These data demonstrated that when rats received sub-motor threshold ES moved around their home cages five times more than when they were not receiving ES, thus demonstrating the possibility to use ES to enable “self-training” in an environment while patients could be performing routine activities of daily living.

The objective of this study was to identify spinal rat’s hind-limb stepping like activity based on EMG patterns and to quantify the effects of multi-modal neuromodulation on spontaneous locomotor activity in standard individual housing cages. We hypothesized that multiple neuromodulatory modalities can transform non-functional spinal networks into more excitable physiological states to enable “self-training” in freely moving spinal rats in their home environments. In addition, motor-evoked potentials (MEPs) observed in hindlimb EMG signals could present a functional biomarker of these novel physiological states (Gad et al., 2015). Spinal rats that had previously been trained to step on a treadmill in presence of Strych and Quip were tested in their home cages under five different conditions, i.e., no ES (Pre), ES, ES + Quip (qES), ES + Strych (sES), and ES + Quip + Strych (sqES). To test these hypotheses, we developed a novel Thresholding Offline Kinematic and EMG Data Analysis method (TOKEDA) to characterize motor behaviors to neuromodulatory interventions. TOKEDA has the capability to be scaled up or down with respect to its sensitivity to detect multiple movement and electrophysiological events during spontaneous activity under different environments.

## Materials and Methods

### Animal Preparation and Care

Data were obtained from four adult female Sprague Dawley rats (270–300 g body weight). Pre and post-surgical animal care procedures have been described previously (Roy et al., 1992). The rats were housed individually in cages with food and water provided *ad libitum*. All survival surgical procedures were conducted under aseptic conditions and with the rats deeply anesthetized with isoflurane gas (1.5–2%) administered via facemask. All procedures described below are in accordance with the National Institute of Health Guide for the Care and Use of Laboratory Animals and approved by the Animal Research Committee at UCLA.

### Head Connector and Intramuscular EMG Electrode Implantation

A small incision was made at the midline of the skull. The muscles and fascia were retracted laterally, small grooves were made in the skull with a scalpel, and the skull was dried thoroughly. Two amphenol head connectors with Teflon-coated stainless steel wires (AS 632, Cooner Wire, Chatsworth, CA, United States) were securely attached to the skull with screws and dental cement as described previously (Ichiyama et al., 2008a). The tibialis anterior (TA, ankle flexor) and soleus (Sol) (ankle extensor) muscles were implanted bilaterally with intramuscular EMG recording electrodes (Roy et al., 1992). Skin and fascial incisions were made to expose the belly of each muscle. Two wires extending from the skull-mounted connector were routed subcutaneously to each muscle. The wires were inserted into the muscle belly with a 23-gauge needle, and a small notch (0.5–1.0 mm) was removed from the insulation of each wire to expose the conductor and form the recording electrodes. The wires were secured within the belly of each muscle via a suture on the wire at its entrance into and exit from the muscle belly. The proper placement of the electrodes was verified during the surgery by stimulation through the head connector and postmortem via dissection.

### Spinal Cord Transection and Electrode Implantation Procedures and Post-surgical Animal Care

A partial laminectomy was performed to expose the T8–T9 spinal cord, and then a complete spinal cord transection to include the dura was performed with microscissors. Two surgeons verified the completeness of the transection by lifting the cut ends of the spinal cord with fine forceps and passing a glass probe through the lesion site. Gel foam was inserted into the gap created by the transection as a coagulant and to separate the cut ends of the spinal cord. For epidural electrode implantation, partial laminectomies were performed to expose the spinal cord levels L2 and S1. Two Teflon-coated stainless steel wires from the head connector were passed under the spinous processes and above the dura mater of the remaining vertebrae between the partial laminectomy sites. After a small portion (∼1-mm notch) of the Teflon coating was removed and the conductor was exposed on the surface facing the spinal cord, the electrodes were sutured to the dura mater at the midline of the spinal cord above and below the electrode sites with 8.0 Ethilon suture (Ethicon, New Brunswick, NJ, United States). Two common ground (indifferent EMG and ES) wires (∼1 cm of the Teflon removed distally) were inserted subcutaneously in the mid-back region on the right side (EMG) and midline (ES) close to the tail. All wires (for both EMG and ES) were coiled in the back region to provide stress relief. All incision areas were irrigated liberally with warm, sterile saline. All surgical sites were closed in layers with 5.0 Vicryl (Ethicon, New Brunswick, NJ, United States) for all muscle and connective tissue layers and for the skin incisions in the hind-limbs and 5.0 Ethilon for the back skin incision. Buprenex (0.01–0.05 mg/kg s every 8–12 h) was used to provide analgesia. Analgesics were initiated before completion of the surgery and continued for a minimum of 2 days. The rats were allowed to recover fully from anesthesia in an incubator. The rats were housed individually in cages that had ample CareFresh bedding, and the bladders of the spinal rats were expressed manually three times daily for the first 2 weeks after surgery and two times daily thereafter. The hind-limbs of the spinal rats were moved passively through a full range of motion once per day to maintain joint mobility. All of these procedures have been described in detail previously (Courtine et al., 2009).

### Training Procedures

All rats were trained for bipedal stepping and standing on a motor driven rodent body weight supporting treadmill for 5 days/week, 20 min/day for 6 weeks starting at 12 days post-injury (dpi), including the days of testing (de Leon et al., 2002). Bipolar ES between L2 and S1 (current flowing from L2 to S1) at frequency of 40 Hz, pulse width 0.2 ms was used in combination with Quip (0.3 mg/kg; de Leon et al., 1999) and Strych (0.5 mg/kg; Gad et al., 2013a) injected intraperitoneally 10 min before each training/testing session. ES was only delivered during the 20 min/day training periods as described previously (Ichiyama et al., 2005). Chronic step training was used to train and reinforce locomotor neural networks that generate spontaneous cage activity (Gad et al., 2013a). At the early stages of training, ES intensity was set at threshold or at super-threshold to invoke locomotive activity. As training continued, the stimulation intensity was gradually reduced, dependent upon the stepping performance of each rat until the stimulation intensity of below threshold.

### Stimulation and Testing Procedures

**FIGURE 1:**
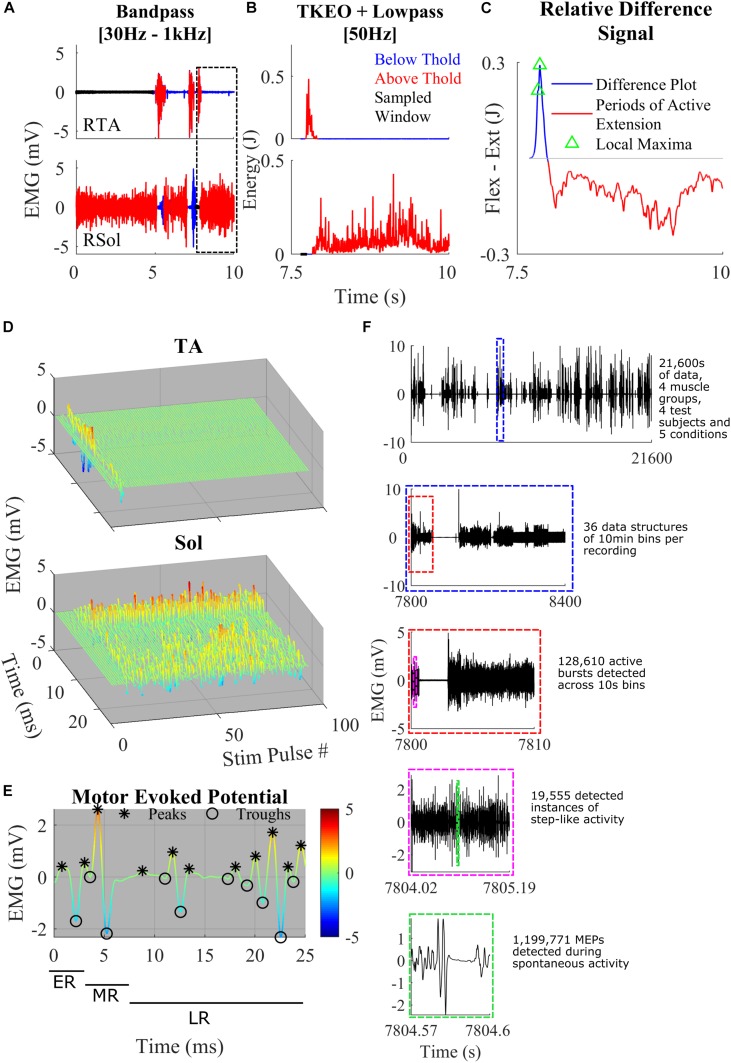
**(A)** Representative band-pass filtering of TA and Sol. The boxed area shows a close-up example of the thresholding and the normalized difference plot used to characterize step-like activity. Regions of the signal below threshold (blue), above threshold (red), and rest (black) were recorded. **(B)** TKEO signal conditioned, rectified, and smoothed EMG signal from the TA and Sol muscle of a spinal rat during spontaneous cage activity. A representative example of a singular hind-limb step within the first hour of recording spontaneous activity. **(C)** The relative difference between the normalized values of the TA and Sol from graphs **(B)**. The gray line *y*-axis = 0. The blue line is a trace of the relative difference plot and in red is the periods of active extension, identified through thresholding. Green triangles represent points of local maxima during active flexion. These features were used to detect spontaneous hind-limb step-like activity. **(D)** A singular identified step using the TOKEDA method as summarized in graphs **A–C**. This plot illustrates motor evoked potentials (MEPs) over time for the tibialis anterior (TA) and Soleus (Sol), respectively. **(E)** A representative MEP from the TA recording shown in panel **D**. Color scaling reflects amplitude in mV. Note the windows marking the early, middle, and late responses and peaks (^∗^) and troughs (o). **(F)** A breakdown of the volume of data involved within the experiments and the process of data structure and analysis. Out of four possible channels, displayed in the top-most subplot is the full 6 h EMG recording of RSol from a singular treatment. Highlighted within the dashed blue box is a 10-min section in which the total hour was sectioned to give a total of 36 files per 6 h recording. The dashed red line bins the 10-min files for thresholding, burst detection, and step detection. The dashed magenta box represents a singular instance of step-like activity from the beginning of swing to the end of the stance phase. Within the dashed green box is a singular MEP measured within a singular instance of the step-like event.

The threshold for eliciting muscle twitch and corresponding time linked EMG response in the Sol was identified. The sub-threshold level was set to 20% below the motor threshold during the recording of spontaneous cage activity. ES was delivered only during the training and testing periods. Each rat prior to testing was injected intraperitoneally 10 min prior to beginning the testing, with the prescribed neuromodulating pharmacology. The spontaneous activity of the spinal rats was determined in their home cage. Spinal rats that had previously been trained to step on a treadmill in presence of Strych and Quip were tested under five different conditions, i.e., no ES (Pre), ES, ES + Quip (qES), ES + Strych (sES), and ES + Quip + Strych (sqES). The head connector was coupled to a set of amplifiers and a stimulator. The swivel arrangement was attached to allow the rats to roam freely in the cage. There was food distributed throughout the cage floor to encourage movement and exploration. IR video data were recorded using a camcorder for select conditions. EMG data were amplified and recorded using custom LabView-based data acquisition software with a sampling frequency of 10 kHz (Figure 1F). Data were recorded continuously for 6 h between 8 p.m. and 2 a.m. One out of the 20 experiments (*n* = 4 rats, five experiments/rat) were conducted every night and were randomized with at least 1 day gap between two successive recordings for a rat and all 20 experiments were completed within 3 weeks. Note that the first 20 min EMG recordings for rat #1 in the sqES and all recording channels for rats #2 and #4 for the ES only case were corrupted and unable to be used.

### Signal Processing

The raw EMG signal was filtered with a third order Butterworth bandpass filter with cut-off frequencies of 30–1000 Hz (Figure 1A). This attenuates DC offset and higher frequency noise. To make the signal more sensitive to change, the bandpass filtered signal was fed into a Teiger Keiser Energy Operator (TKEO) algorithm (Solnik et al., 2010). The output was rectified and smoothened across a 50-Hz cut-off low pass filter (Figure 1B).

### Thresholding

To determine the threshold of activity across each channel, the 6 h recordings were broken up in to 10 s bins (Figure 1F). A threshold sampling method was used to determine periods of rest throughout each bin. To find periods of rest, the data were averaged across 10 ms and the minimum was selected as the first point of interest. A sliding window procedure was implemented to approximate the entire period of rest within the 10 s bin. This was limited by a first differential magnitude of 0.1 mV/s or logarithmic threshold of 300 ln (mV). It was found that during periods of slower, more gradual ramping EMG activity, the first and second differential values were not sufficiently sensitive. To account for slow ramping activity, the logarithmic threshold was implemented. This enables the software to detect both quick and slower adapting changes across the EMG signal.

A double thresholding mechanism was used to detect bursts of EMG. The mean and standard deviation of the detected rest period was calculated and used to determine a threshold for EMG magnitude. Given the circumstance of recording and filtering, a scaling constant (*J*) of 7 was used. Finally, to complete the double thresholding, a time criterion was implemented for each channel, where the TA channel time threshold was 0.1 s and Sol channel time threshold was 0.3 s. If the signal magnitude was below the time threshold, it was not considered bursting activity. The minimum criteria for an EMG magnitude to remain above the motor threshold as defined by:

$$\text{threshold}={\text{μ}}_{\text{rest}}+J^{*}\,\sigma_{\text{rest}}$$

For an example of a period of an EMG signal which met the magnitude threshold but not the time criteria, see the RSol channel of Figure 1A.

### Activity Detection

To detect instances of step-like behavior, a dataset was generated using IR video data. A total of 26.7 h of video data was collected. Step-like, standing, and rest events were compiled into a dataset of activity. These data were then synchronized with EMG recordings of the TA and Sol of both left and right hind-limbs to produce 300 EMG samples from a combined total of 217 step-like events and 83 non-functional activity events. While there were several standing events detected throughout the 6 h period, analyses were limited to step-like events only.

A relative difference signal between a normalized TA and Sol recording was calculated to determine instances of spontaneous step-like activity (Figure 1C). The TA and Sol signals were normalized to account for missing force-EMG measurements. This signal was used as a representation of coordination between extension and flexion of the ankle. It was assumed that if the signal was positive, the ankle was applying a greater flexion force on the joint compared to extension, vice versa for negative magnitudes. A peak during the positive region of a signal followed by a sharp dip into the negative portion of the signal was associated with the reduction in flexion activity during swing phase and an activation of extensors during the stance phase. Peaks from the relative difference signal were only considered if they were positive in magnitude and during an active TA EMG burst. By using the active burst regions as a “filter,” many false positives of locomotion initiation were removed.

For conservative activity detection, a step was only registered when a positive peak was followed by a negative signal during an active extension EMG burst achieved within a threshold of 0.5 s. Only peaks with a magnitude greater than the difference threshold of 0.01, in the relative difference signal and only magnitude differences greater than the difference threshold were considered. The time and magnitude thresholds were characterized from numerous test examples in the given dataset. In order to filter coordination and reflect false positives, a time threshold for active Sol burst was characterized at 0.25 s. Finally, a minimum period between each local maxima of 0.2 s was set. This was to ensure that only the last local maxima within the TA burst was selected.

Accuracy was defined as the ratio between correctly detected events over the total number of sampled events. Precision was defined as the number of true positives out of both true and false positives; thus, an estimate of the number of actual step-like events detected. Recall was defined as the number of true positives out of false negatives and true positives; thus, an estimate for detecting all relevant step-like events within the existing dataset.

**FIGURE 2:**
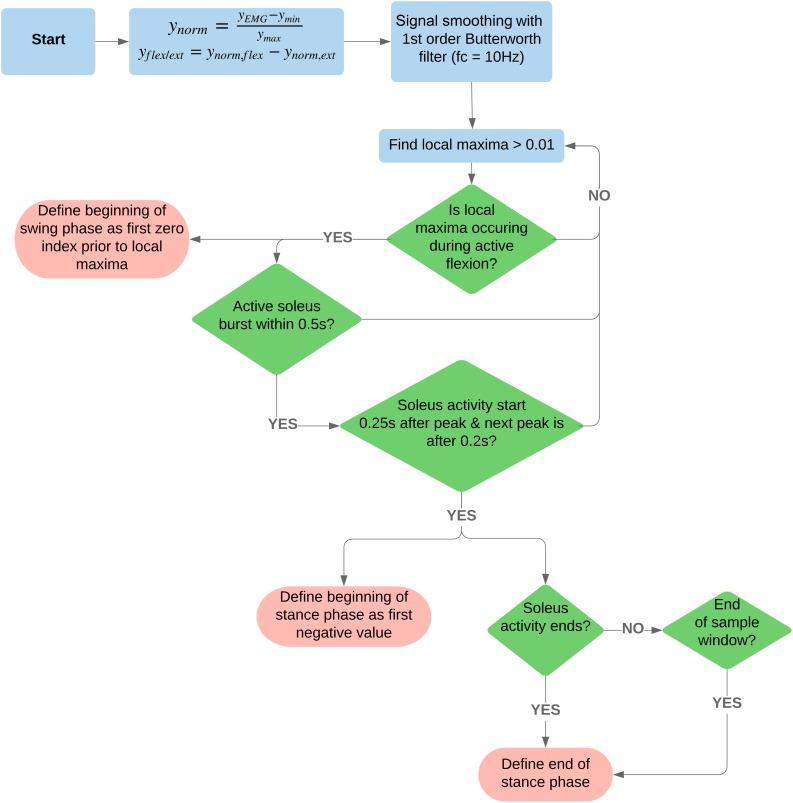
Flowchart of the signal processing and algorithm used to detect beginning and end of swing and stance phases. For access to the code, see the following GitLab repository, https://code.research.uts.edu.au/13035516/tokeda.git.

For a complete algorithm process illustration, refer to Figure 2.

### MEP Analysis

For granular electrophysiological analysis, the entire step cycle was defined from the beginning of the swing phase to the end of the stance phase. This was set at the point where the relative difference signal first reaches zero before the detected peak and where the negative signal reaches zero or the extension EMG burst has ended (Figure 1C). With these defined windows of step-like activity, MEPs were analyzed to extract electrophysiological features such as the number of positive peaks and integrated EMG for both the middle and late response (LR) (Figures 1D,E). The MEPs were extracted from the bandpass-filtered EMG data. Using a recording of the stimulation pulses, the end of each 40 Hz stimulation pulse was used to separate the EMG signal into 25 ms MEPs. The early response (ER), middle response (MR), and LR time windows were defined as 1–4, 4–7, and 7–25 ms, respectively (Figure 1E). To successfully detect the peaks and troughs of the MEP signal, a threshold using the second differential was used. Each MEP was interpolated to double the number of points. Using the interpolated signal, the local maxima and local minima with a minimum separation of 0.6 ms and minimum prominence of 0.2 were found. These local maxima and minima were filtered through a second differential threshold of 1.5 × 10^6^ such that only peaks and troughs that met the threshold criteria were accepted (Figure 1C). This was used to calculate the area under the curve (IEMG), peak-to-peak, and total number of peaks.

### Statistical Analyses

All data reported as means ± standard error (SE). Statistically significant differences were determined using a one-way ANOVA with the Tukey–Kramer *post hoc* test, correlation coefficients were calculated using Pearson’s linear coefficient. All statistical difference was set at *P* < 0.05. All results reporting step-like activity were normalized to no intervention (Pre) measurements.

## Results

### Task Recognition Validity

To test the validity of the task recognition algorithm derived from the relative difference signal, the point of change from flexion phase to extension phase during a “step-like” event was logged (difference signal equals zero). A dataset of IR video and EMG-derived step-like examples were collected to form the human-observation-based dataset. In this dataset, the onset and completion of a step-like or rest event for each limb was logged to a millisecond precision. The human-observation dataset comprised of 300 EMG samples from a combined total of 217 step-like events and 83 non-functional activity events. A validation script checked the algorithm event log against the human-observation dataset. To gather a sense of relevance in detected step-like patterns, accuracy, precision, and recall were calculated from the validation script. The relative difference signal detected step-like activity performed with an accuracy of 83%, precision of 88%, and recall of 89%.

### Electropharmacological Treatments Modulate the Functional State of Spinal Circuitry

**FIGURE 3:**
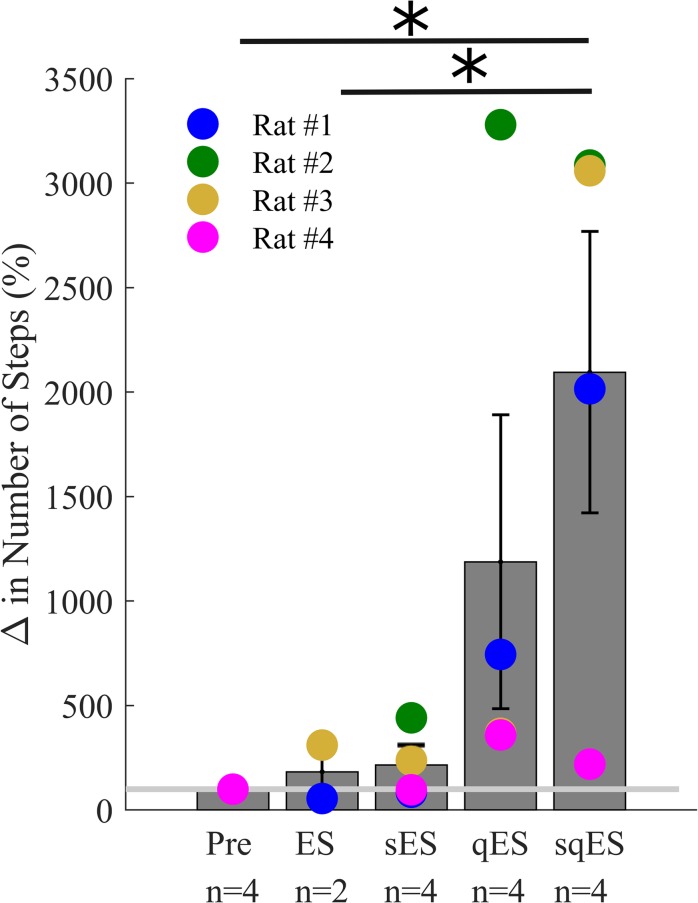
Mean% change in step-like events for each rat over the 6-h period for each treatment (^∗^). The light gray line is set at 100%, normalized to pre-treatment for each rat. Results for sqES were significantly different compared to Pre and ES (*P* < 0.05).

All interventional conditions increased the number of spontaneous step-like activity compared to baseline (Pre-intervention) (Figure 3). Quip or Strych significantly increased the number of recorded step-like activity in all animals tested. A significant level of variability was observed among rats, especially in the qES and sqES experiments. However, sqES treatment generated the highest number of steps recorded when compared to Pre and ES (*P* < 0.05).

**FIGURE 4:**
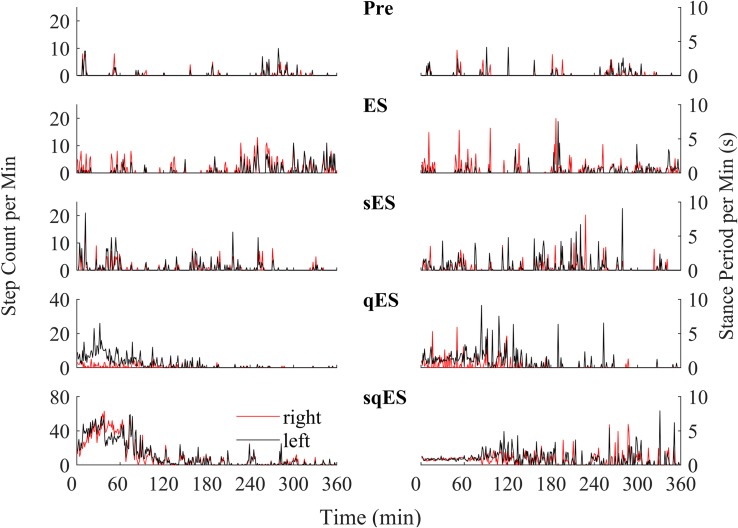
An example of the step-like activity across interventions plotted as number of steps per minute and mean stance periods for the **left** (black) and **right** (red) hind limb for rat #3. For other rat performances, refer to Supplementary Figures 1–3.

**FIGURE 5:**
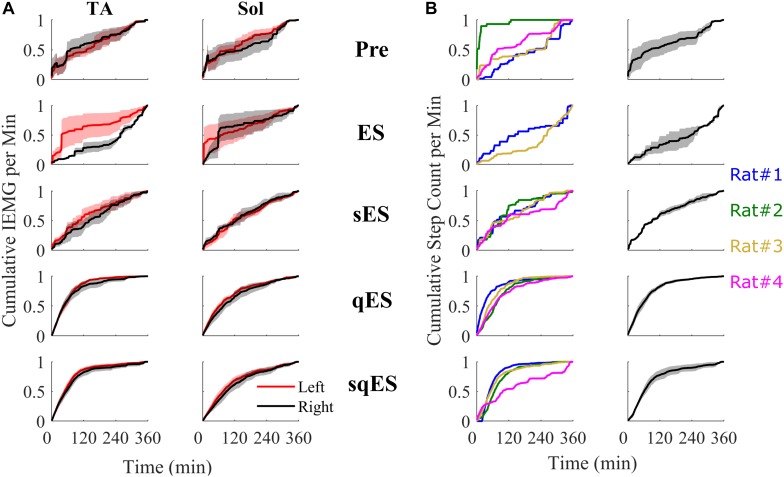
All cumulative sum plots are normalized per animal from 0 to 1. **(A)** Cumulative sum of IEMG in of the left and right TA and Sol. **(B)** On the left is a cumulative sum plot of the step-like events occurring over time for each treatment (blue, red, gold, and magenta represents rat #1, #2, #3, and #4, respectively). On the right is the averaged cumulative sum plot. Shaded is the standard error.

Since a single bolus dose of a pharmacological treatment was administered at the start of the experiment, we observed a time-dependent phenomenon in the number of steps detected with the greatest level of activity observed in the first 120 min (Figure 4). These observations were specific to the rapid registration of step-like activity for sqES and qES treatments (Figure 5B). This time-dependent phenomenon was not observed in Pre or ES cases. The stepping activity occurred stochastically throughout the 6 h (Figures 4, 5B). Spontaneous step-like activity during the first 120 min occurred with longer, more consistent step lengths and increased muscle activity during sqES and qES (Figures 4, 5A,B). During continuous stepping activity, an average stance period of ∼2 s was observed (Figure 4). Both the step activity and stance period across the population had lower variability during sqES compared to other conditions tested (Figures 4, 5B). A smaller variance was present in the sqES stance period compared to qES within the first 2 h. In addition, during the entire 6-h period, overall left–right symmetry was maintained suggesting a bipedal response (Figure 4).

**FIGURE 6:**
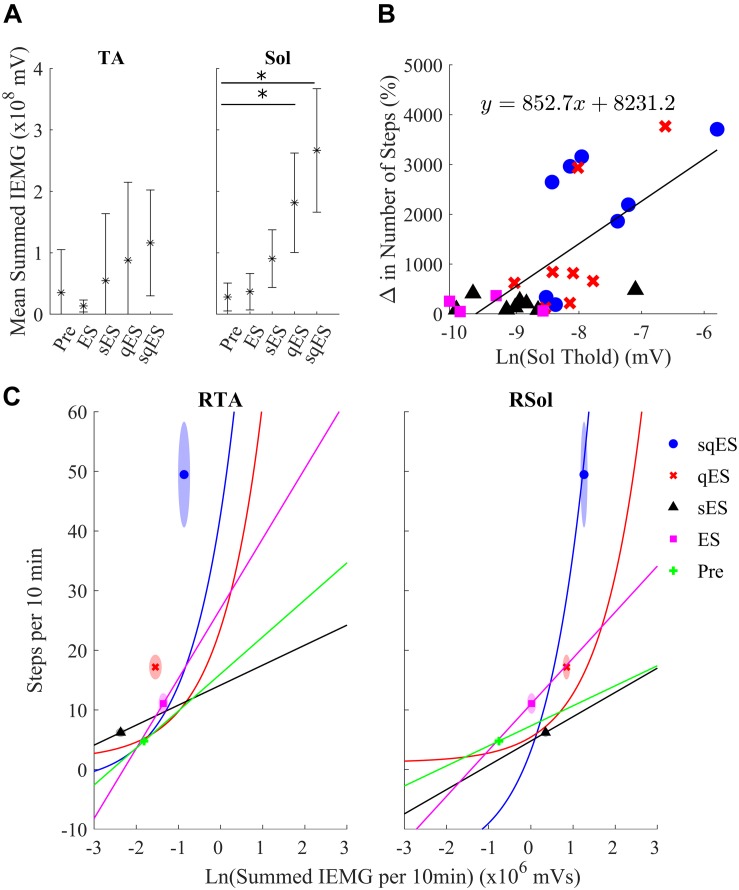
**(A)** The average sum of IEMG across all the animals in each treatment in the TA and Sol channels for both sides and the 95% confidence interval. The Sol channel for sqES and qES was significantly different from Pre (^∗^*P* < 0.05). There is a trend of increasing mean and standard error in IEMG in both TA and Sol as more interventions are introduced on top of ES. **(B)** The graph depicts the relationship of the calculated EMG amplitude threshold required to determine burst activity of the neural networks in the Sol and the change in step-like activity normalized to pre intervention for all rat subjects in both hindlimbs. The treatments, sqES, qES, sES, and ES are plotted in blue, red, black, and magenta, respectively. A linear regression of the natural logarithm of Sol threshold values against the normalized change in step-like activity results in *R* = 0.6943. Inclusion of pharmacology and ES increases the ln(Sol) threshold, only qES and sqES have drastic change in detected step-like events. **(C)** Ln(Total IEMG/10 min) plotted against step-like events/10 min across 6 h for sqES, qES, sES, ES, and Pre in the right hind-limb. In the corresponding shaded area is the standard error for the *x*- and *y*-axis represented in an ellipse. A Pearson’s linear correlation coefficient reveals significant positive correlation in all interventions for both hind-limbs (*P* < 0.05 in all cases). While the sES, ES, and Pre interventions are linearly correlated, the qES and sqES have an exponential relationship between the natural log of the total IEMG/10 min and the step-like events/10 min. Note that sqES, qES, and ES have a distinct *y*-axis error bound while the *x*-axis error bound is less notable. This is especially notable in the RSol graph.

Inclusion of multiple pharmacological agents in the presence of ES resulted in a more consistent and greater IEMG response (Figure 5A). While sES reduced the variability across both extensor and flexor muscles in the hindlimbs, the inclusion of Quip significantly increased IEMG magnitude compared to Pre (*P* < 0.05) (Figures 5A, 6A). For the ES and Pre treatments, the IEMG of TA differed in shape compared to Sol with wider error bands while the sES, qES, and sqES relatively coordinated activity in the TA and Sol muscles with narrower error bands were observed (Figure 5A). Differences between TA and Sol may be explained by the higher occurrence of co-contractions and organized coordinated activity during the pharmacological interventions. The steep gradient was only observed during the first 2 h of sqES and qES and becomes more linear during hours 2–6, possibly indicating the effective half-life of the drug. Significant correlation was observed between the overall muscle activity (IEMG) and the steps registered across both right hind-limb muscles (Figure 6C, *P* < 0.05). Both sqES and qES have an exponential relationship between IEMG and step-like activity, while sES, ES, and Pre reflect a linear correlation. sqES intervention had the largest SE across both the *x*- and *y*-axes of Figure 6C whereas sES and Pre have minimally discernible spread among the sampled population. Increased baseline tone in Sol during rest correlates (*R* = 0.6943, *P* < 0.05) with the increased step-like activity across all conditions and rats and consistent with the overall summed IEMG across the 6 h (Figure 6B). There appears to be a breakaway condition given the logarithmic response in the basal tone in Sol represented (Figure 6B).

### Evoked Responses Correlate With Spontaneous Step-Like Activity

**FIGURE 7:**
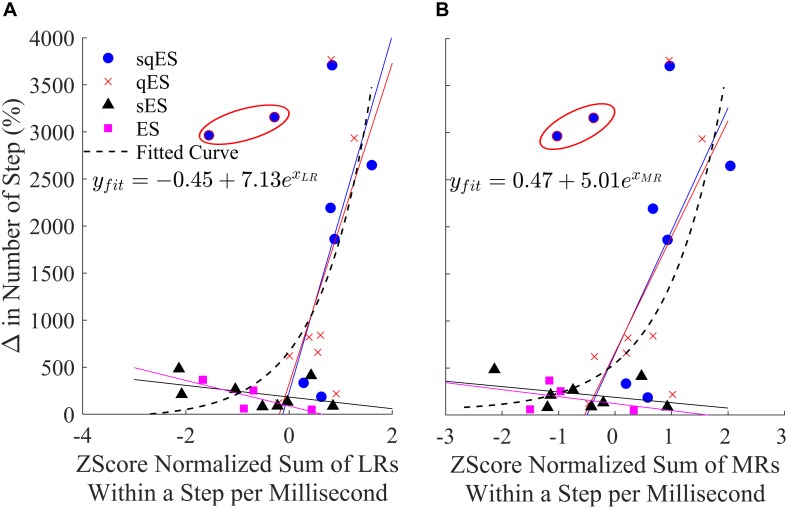
**(A)** Change in the number of late and **(B)** middle peaks responses (normalized to pre values) for each rat, treatment, and left and right hind-limb of the Sol MEPs. Circled in red are two outliers identified as rat #3 during the sqES treatment. Additionally, linear regressions for each of the respective treatments in their corresponding color are plotted. An exponential curve (dashed line) encompasses all treatments.

Motor-evoked potentials were analyzed across the Sol during spontaneous step-like activity as these signals more accurately reflect the state of the neural networks associated with the trained task. The detected peaks in the middle and LRs (MRs and LRs) of the Sol during spontaneous step-like activity in their home cages were exponentially correlated (Figure 7). Due to the sub-threshold nature of spinal stimulation, the evoked responses appeared to be non-time linked to the stimulation pulses when compared to suprathreshold stimulation on a treadmill (Gad et al., 2013b). Each of the treatments had linear regression applied to both the LRs and MRs. Both ES and sES display a negative gradient associative with decreasing change in step-like activity when compared with Pre while qES and sqES have a steep positive gradient. The combination of all data points was fitted to an exponential equation where both the LRs and MRs have a similar relationship with step-like activity, the MR trend has a lower first-order coefficient when compared to the LR. As anticipated, the sqES and qES evoked the highest number of LRs and MRs which corresponded to a greater number of detected spontaneous step-like activity.

## Discussion

We developed a method for identifying spinal rat’s hind-limb stepping like activity based on EMG patterns recorded from the Sol and TA to determine the effects of multi-modal neuromodulation on spontaneous locomotor activity in standard individual housing cages. These results describe the functional and electrophysiological changes in response to activity-specific training in combination with enabling ES and pharmacological interventions. For access to longitudinal functional analyses, refer Gad et al. (2015). This experiment was conducted to determine the feasibility of developing a quantitative electrophysiological assessment tool of spontaneous cage activity of rats as an estimate of neuromuscular activity.

Several strategies have been developed for detecting rat activity within a caged setting using a variety of sensing methods. These include the use of vibration/tilt sensing (Parreno et al., 1985; Megens et al., 1987; Ganea et al., 2007), IR beams (Clarke et al., 1985), IR and non-IR video tracking (Tamborini et al., 1989; Morrel-Samuels and Krauss, 1990; Aragão et al., 2011; Gad et al., 2013a; York et al., 2013), capacitive flooring (Pernold et al., 2019), optical touch sensors (Mendes et al., 2015), RFID (Redfern et al., 2017), and radar technology (Martin and Unwin, 1980; Rose et al., 1985; Young et al., 1996; Genewsky et al., 2017). Most pattern recognition approaches based on EMG recordings have been used to control prosthetics (Huang et al., 2009, 2010). A more complex approach involves the use of Hidden Markov Models (HMMs) to represent stochastic processes using time series data to predict the probability of model states (Lee, 2008). However, the detection of spontaneous self-recovering movement after paralysis outside of a controlled clinical environment is rarely explored quantitatively due to the limitation associated with securely attaching sensors or markers at specific locations, etc. Use of antennas attached to a rat have been used to track gross body movements through a maze of tubes. It was shown that self-motivated training within a “enriched environment” lead to superior performance in skilled movement compared to restricted task-specific training. This “RatTrack” system allows for testing self-initiated and task-specific training and dose–responses (Starkey et al., 2014). While the mentioned efforts can be extended in the direction of automation, each of these methods lack the ability to have a direct measurement of the neuromotor parameters. TOKEDA relies solely on chronically implanted EMG electrodes for sensing to assess *in vivo* responses to different combinations of electrical and pharmacological neuromodulatory interventions. Having access to neuromuscular activity synchronized with stimulation pulses directly linked to behaviors provides a direct and realistic measurements of reorganizing neural networks throughout a chronic period as the nervous system becomes more functional (Edgerton et al., 2008). As the MEPs and associated behavior are directly linked from an “input–output” relationship, we hypothesize that a biomarker for reorganization may be discernible from the presented data. This notion has been reflected in past research papers (Lavrov et al., 2006, 2008; Gad et al., 2015; Alam et al., 2017). Unfortunately, without the use of kinematic recordings, information such as locomotion speed, step quality, step length, and other kinematic-related metrics remain remarkedly rare given the availability of the appropriate technologies.

Long-term recordings of electrophysiological data in laboratory animals and humans before and after treatment for a dysfunction have been performed previously (Alaimo et al., 1984). The existing hypothesis regarding the possibility of electrophysiological biomarkers is the emergence of MRs and LRs in MEPs during activity-driven training (Lavrov et al., 2006, 2008; Gad et al., 2015). There appear to be underlying neurological mechanisms involved in time-related modulation in MEPs during treadmill activities. In Gad et al. (2015), it was shown that re-emergence in LRs and reduced MRs with changes in modulation of flexor extensor motor pools reflect plasticity of the neural networks. Another study reports a correlation between the MR and LR with changes in the EMG activity level of the corresponding muscle, perhaps, reflecting in part monosynaptic and polysynaptic pathways, respectively, to motor pools (Gerasimenko et al., 2006). In the present paper, we explore the characteristics of the same signals during spontaneous *in vivo* step-like activity within an enriched caged environment. Paralleled between these findings, Figure 7 presents an exponentially increasing trend between the emergence of LRs and the functional response to hind-limb step training. Notably, while Gad et al. (2015) concludes a reduced reliance on MRs may indicate a change in targeted neural networks and neuro-plastic mechanisms, Figure 7 illustrates no distinct difference between LRs and MRs beside a slightly lower exponential coefficient. These contrasting reports may be due to the small sample size or due to difference in experimental environment. While Gad et al. (2015) performed experiments within a weight-supported treadmill setting, the presented experiment was conducted in a weight bearing, free roaming environment. This difference in locomotive setting may introduce disruptions to the reorganization of neural networks due to the loss of highly organized and predictable patterns of sensory input that is normally generated with step training on a treadmill (Edgerton and Roy, 2009; Taccola et al., 2018).

Additionally, note that the highlighted outliers in Figure 7 were not included in the regression calculations. These data points suggest that at below average low MEP peak counts, high levels of activity can occur. These outliers were measured from the same rat within the same experiment. During analysis of the video data, an unusual frequency of induced, air-stepping was observed (Supplementary Videos 1, 2). The fact that some number of these counted step-like events were not enabled but rather induced by ES may result in minimal polysynaptic pathways being activated during air-stepping, i.e., when the proprioceptive and tactile afferents typically associated in over-ground, weight-bearing hind-limb locomotion were not present. A number of possible reasons could provide explanation as to why repetitive, non-weight bearing cycles could readily occur.

The exponential trend visible in Figure 7 alongside the data in Figures 6B,C present the possibility of a “breakaway” point where the minimum level of neuronal activity and polysynaptic mechanisms is required for significant functional improvement. It was only when Quip was introduced where a significant improvement in spontaneous step-like activity was observed. Given the extensive research provided with training under the influence of Strych, one would expect that Strych experiments would provide equal benefit when compared with Quip (de Leon et al., 1999; Gad et al., 2013b, 2015).

Albeit a small sample size, these data extend the hypothesis of the existence of electrophysiological biomarkers and warrant the extension of further investigation into longer term longitudinal studies and the involvement of more robust algorithms and *in vivo* sensing technologies. These observations alongside the previously discussed representation of plastic mechanisms in MEPs agree with our initial hypothesis of multiple neuromodulatory modalities transforming non-functional spinal networks to a more excitable state to enable “self-training.”

Presented here is a novel method of simultaneously identifying step-like activity while recording electrophysiological biomarkers of stepping. The value of these types of data can be further improved with the introduction of additional sensors in order to compose a more comprehensive assessment of movements as occurs *in vivo* in a variety of environments. As gait patterns have a state-specific nature, repeating the task recognition exercise using a stochastic probability based method such as HMMs (Byung-Woo et al., 1997; Sy Bor et al., 2006; Naeem and Bigham, 2007; Lee, 2008) or long short-term memory (LSTM) (How et al., 2014; Tsironi et al., 2017) with the existing datasets may improve the overall accuracy of the task recognition. The present data provide an example of a “proof of concept” approach to examine the level of activity dependence that is present when testing the efficacy of an intervention. It is technically becoming more feasible to chronically record EMG from many muscles to detect how the reorganization of neuronal networks that control locomotion can be focused on the patterns of coordination of flexion/extension, abduction/adduction, and non-repetitive tasks such as grip and pinch maneuvers as well as repetitive tasks such as cycling, etc. Moreover, the ability to measure the state of the locomotive neural circuitry to determine the direct relationship between the treatment provided and the underlying neuronal mechanics serves as detailed insight as the subject undergoes training, providing the opportunity to adjust treatments in order to maintain an enabling effect, maximizing activity-dependent recovery.

In addition, our algorithm was used to examine the effect of pharmacology in combination with ES by directly comparing the occurrence of step-like activity within a chronic self-training environment. The ability to record chronically for 6 h in a natural environment highlights the functional and electrophysiological changes over time and could represent a functional biomarker of recovery post paralysis. This leads to a potentially critical question in the rehabilitation strategy post paralysis. Can daily periods of sub-threshold electrical neuromodulation of the spinal circuitry enhance the performance of motor skills as well as or even more than when a task-specific rehabilitation paradigm is imposed? If that is the case, then the results will have an immediate impact on how neuromodulation can be used to enhance sensorimotor recovery in individuals with other neural disorders than SCI. Spinal circuits controlling stepping and standing (locomotion and posture) can be improved after a SCI by practicing those tasks, i.e., by increasing the activation of those circuits (Ahissar and Hochstein, 1997; Bayona et al., 2005). The data presented here demonstrate that there is a minimal amount of spontaneous activity in the sensorimotor circuits that control standing and stepping after a mid-thoracic spinal cord transection in adult rats (Figures 4, 5) that can be further enhanced using multimodal neuromodulatory mechanisms. In effect this enhanced level of spontaneous activity can be viewed as a “self-training” phenomenon. Clinically, the presence of a “self-training” effect would have an immediate and significant impact on designing rehabilitation strategies.

While the presented data are based on data derived from spinally injured rats that have had multiple training sessions under the influence of ES and pharmacological agents, longitudinal functional data and analysis can be found in the paper presented previously (Gad et al., 2015). Longitudinal functional analyses were performed at 12, 22, 35, and 49 days past injury with hindlimb joint kinematics tracked with EMG recordings of the TA and Sol under ES, qES, sES, and sqES conditions during standing and step training in a weight supported treadmill. Future work should include continual measurements of functional neurological changes as reported here, but throughout a training period. The present data are largely focused on the acute effects of spontaneous activity after completion of electrical and pharmacological neuromodulation which facilitated self-training (Figure 4). A repetition of this experiment with a larger sample size and with a regimented level of a specific motor task would be of significant interest. Adaptation of the existing algorithm with machine-learning algorithms such as HMMs and LSTM could further enhance our understanding of the more critical variables intrinsic to the mechanisms of activity dependent modulation of neuromotor facilitated reorganization.

## Conclusion

We successfully demonstrated the proof of concept algorithm to detect specific functional hind-limb spontaneous locomotion using minimal biosignals while monitoring biomarkers that represent a polysynaptic neurophysiological system input–output response of the spinal circuitry that controls locomotive activity. In addition, we observed a correlation between these biomarkers and the functional responses to spinally evoked potentials. By incorporating complex machine-learning methods and measuring kinematic output, the algorithm developed can be readily scaled to detect more complex tasks.

## Data Availability Statement

The datasets generated for this study are available on request to the corresponding author.

## Ethics Statement

The animal study was reviewed and approved by the Animal Research Committee at UCLA. All procedures described are in accordance with the National Institute of Health Guide for the Care and Use of Laboratory Animals.

## Author Contributions

RC developed the algorithm and performed the data analysis. PG performed the experiments and consulted in data analysis. HZ performed the surgeries. RC, PG, and VE interpreted the data. RC, HZ, PG, BV, and VE wrote the manuscript. All authors read and approved the final manuscript.

## Conflict of Interest

VE holds shareholder interest in NeuroRecovery Technologies and holds certain inventorship rights on intellectual property licensed by The Regents of the University of California to NeuroRecovery Technologies and its subsidiaries. PG and VE hold shareholder interest in SpineX Inc.

The remaining authors declare that the research was conducted in the absence of any commercial or financial relationships that could be construed as a potential conflict of interest.
