# Supplementary material for: Novel Activity Detection Algorithm to Characterize Spontaneous Stepping During Multimodal Spinal Neuromodulation After Mid-Thoracic Spinal Cord Injury in Rats
Source: Front Syst Neurosci. 2020 Jan 15;13:82. doi: 10.3389/fnsys.2019.00082 (PMC6974470; doi:10.3389/fnsys.2019.00082)
Supplement: Supplementary file 1 [file Data_Sheet_1.docx]

Supplementary Material

# Supplementary Figures


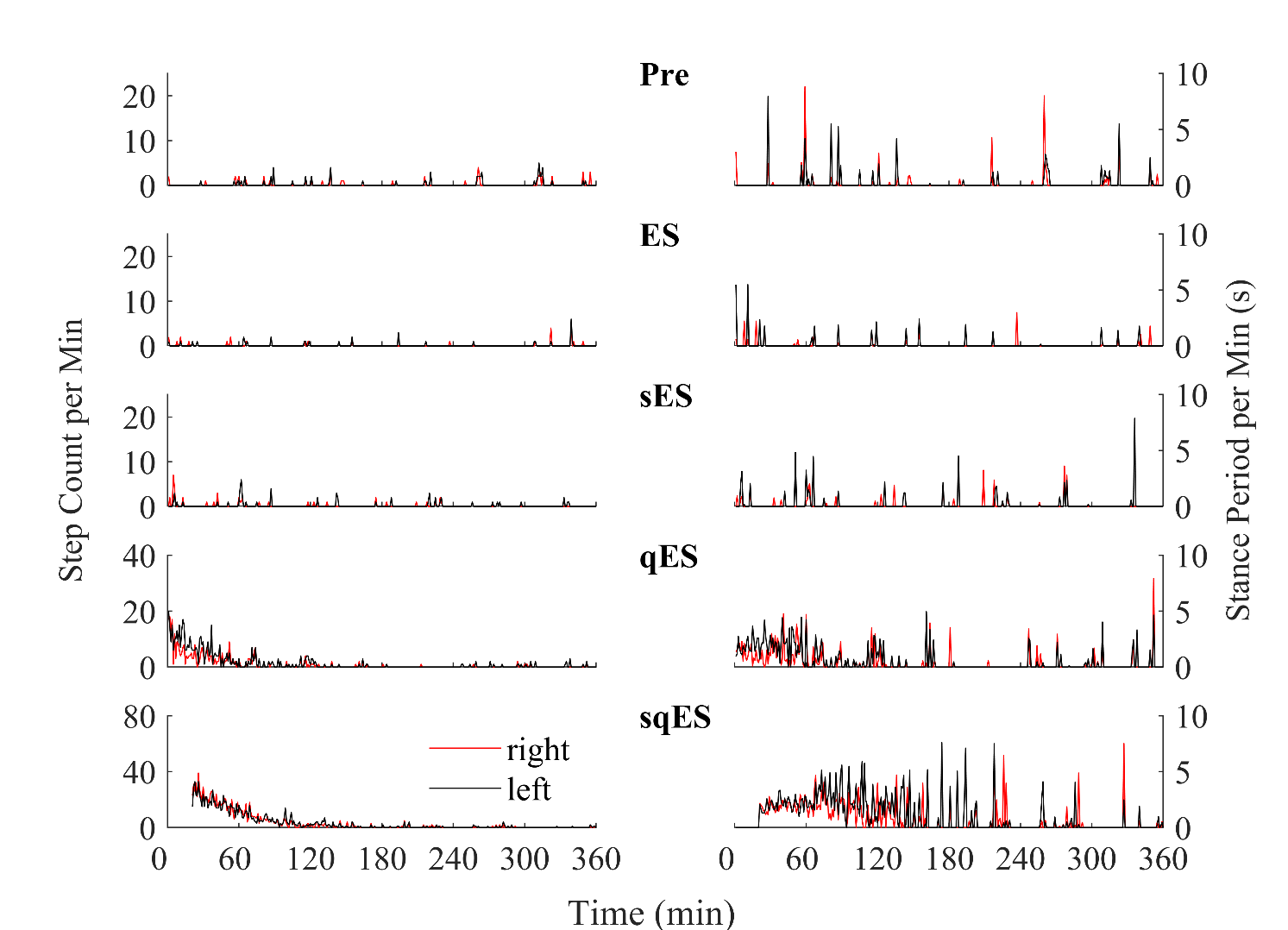


**Supplementary Figure 1.** An example representation of the functional performance across interventions plotted as number of steps per minute and mean stance periods per minute throughout the 6 hours of recording for the left (black) and right (red) hind limbs for rat #1.


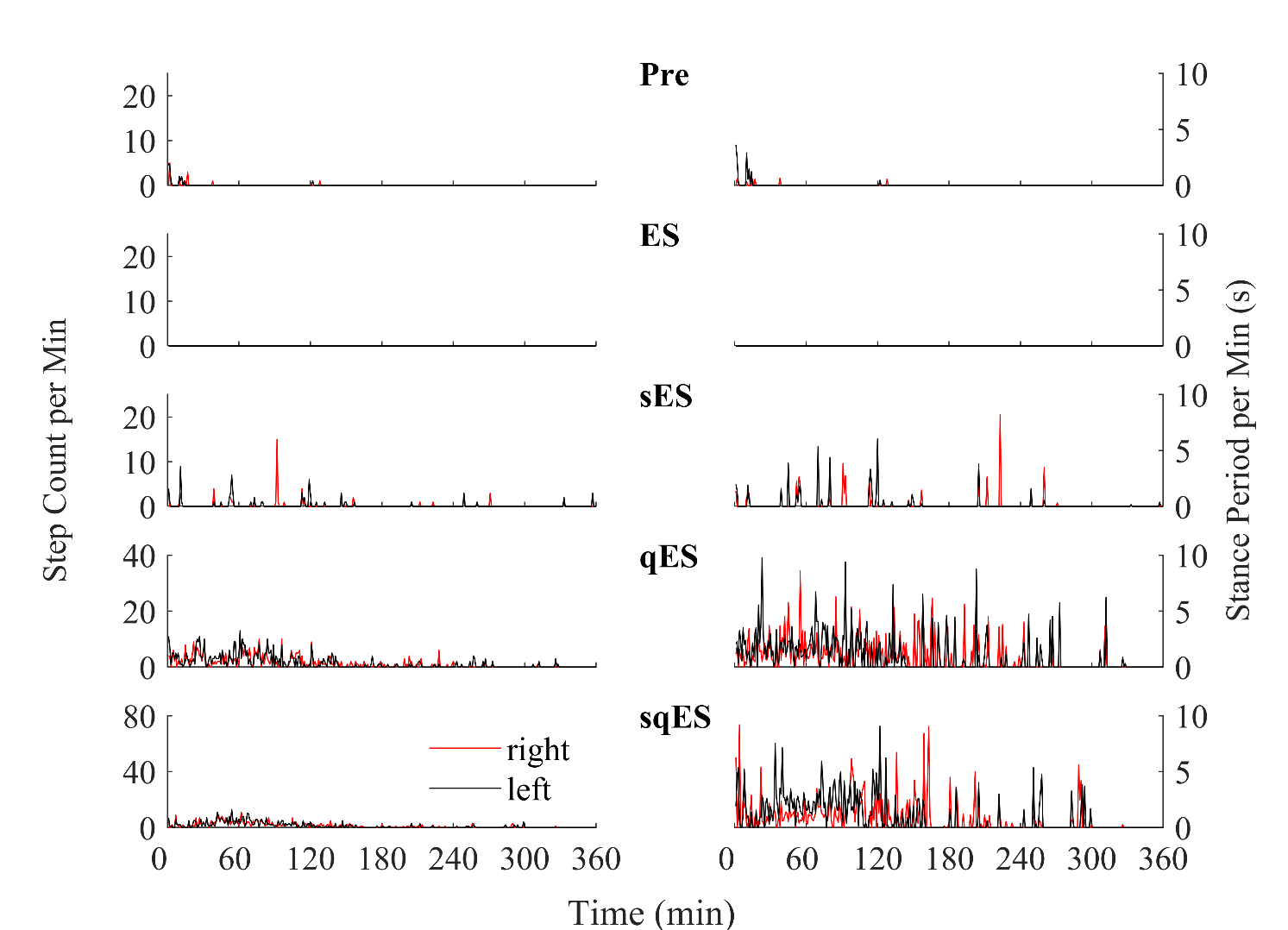


**Supplementary Figure 2.** An example representation of the functional performance across interventions plotted as number of steps per minute and mean stance periods per minute throughout the 6 hours of recording for the left (black) and right (red) hind limbs for rat #2.


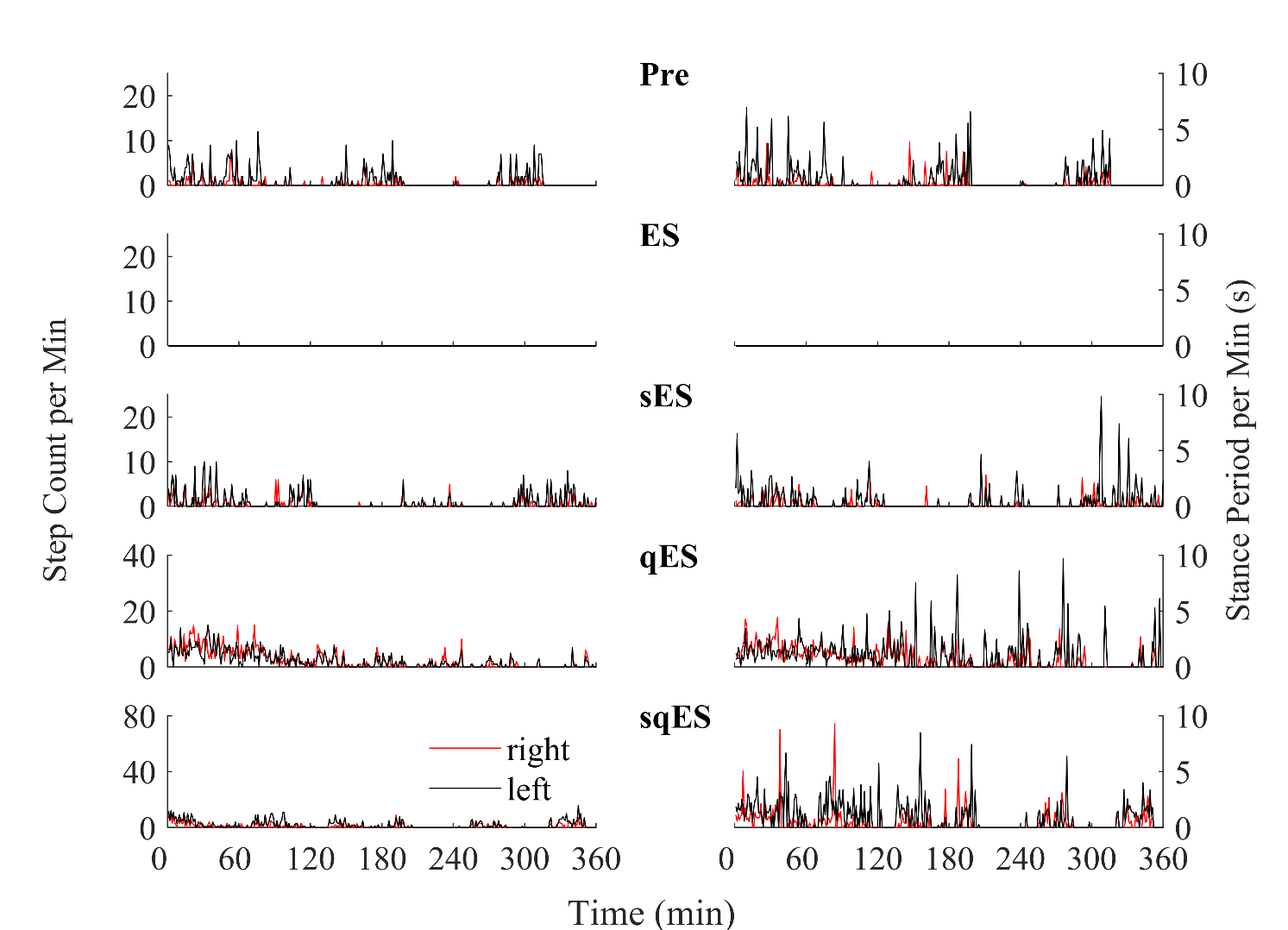


**Supplementary Figure 3.** An example representation of the functional performance across interventions plotted as number of steps per minute and mean stance periods per minute throughout the 6 hours of recording for the left (black) and right (red) hind limbs for rat #4.

# Supplementary Videos

**Supplementary Video 1.** Representative animal with sqES demonstrating air-stepping.

**Supplementary Video 2.** Representative animal with sqES demonstrating alternating hind-limb step-like activity during forelimb driven locomotion and bipedal standing while weight bearing.
